# A State-of-the-Art Review of Ophthalmological Indications for a Cesarean Section: Is There a Patient for Whom a Cesarean Section Is Really Indicated?

**DOI:** 10.3390/diagnostics15040418

**Published:** 2025-02-09

**Authors:** Paola Quaresima, Giuseppe Covello, Giovanna Bitonti, Costantino Di Carlo, Michele Morelli, Maurizio Guido

**Affiliations:** 1 Department of Obstetrics and Gynecology, Azienda Sanitaria Provinciale di Cosenza, 87100 Cosenza, Italy; 2 Department of Surgical, Medical, Molecular Pathology and of Critical Area, University of Pisa, 56126 Pisa, Italy; giucovello@gmail.com; 3 Department of Obstetrics and Gynecology, “SS. Annunziata” Hospital, 87100 Cosenza, Italy; giovanna.bitonti@gmail.com; 4 Department of Public Health, University of Naples Federico II, 80134 Naples, Italy; cdicarlo@unina.it; 5 Department of Pharmacy, Health and Nutritional Sciences, University of Calabria, 87036 Rende, Italy; morellimichele122@gmail.com (M.M.); maurizioguido@libero.it (M.G.)

**Keywords:** cesarean section, myopia, retinal detachment, diabetic retinopathy, glaucoma, vaginal delivery

## Abstract

**Purpose:** Our purpose was to review the current literature regarding ophthalmologic indications for cesarean section (CS). **Methods:** A literature search was conducted using MEDLINE, Embase, and the Cochrane Library from inception through October 2024. The databases were searched using the following keywords: “Caesarean section” OR “Caesarean section” OR “delivery” OR “pregnancy” AND “eyes” OR “eye disorders” OR “ocular disease” OR “diabetic retinopathy” OR “myopia” OR “retinal detachment” OR “glaucoma” OR “keratoconus”. Studies were considered eligible if they described pregnancy management in women affected by an eye disorder, with insight into the mode of delivery. **Results:** A total of 8383 results were identified, including only 1 specific guideline and no randomized controlled trials. After a manual review, 38 manuscripts were selected for inclusion. Based on the available evidence, an elective CS may be considered on a case-by-case basis in the presence of specific ophthalmic conditions, such as high-grade myopia with subretinal neovascularization, proliferative diabetic retinopathy, advanced glaucoma, or advanced keratoconus. These conditions are rare among women of childbearing age. **Conclusions:** Currently, only a limited number of highly specific ophthalmic conditions may benefit from an elective CS. Considering the potential short- and long-term implications of a CS, and in line with the current World Health Organization recommendations, this surgical procedure should be reserved for cases with a clear indication. Given the paucity of data in the available literature, further prospective randomized controlled trials are necessary to enhance the quality of evidence.

## 1. Introduction

Cesarean section (CS) is a major abdominal surgery associated with immediate maternal and perinatal risks, with potential implications for future pregnancies as well as long-term effects that are still under investigation [1]. Over the years, the global CS rate has significantly increased from around 7% in 1990 to 21% today, surpassing the ideal acceptable CS rate, which is around 10–15%, according to the World Health Organization. This underlines the importance of limiting surgical delivery only to those cases with recognized indications [2]. Among the most common non-obstetric indications for CS, the presence of maternal ophthalmic pathology plays a significant role [3,4,5,6]. Eye disorders are divided into refractive errors, retinopathy, retinal detachment, glaucoma, and keratoconus. Traditionally, many of them have been considered an indication to deliver by CS, aiming to avoid the potential worsening or recurrence of the eye disorder due to the Valsalva maneuver during labor [3]. Moreover, clinicians have been more inclined toward surgical delivery due to the fear of litigation in obstetric care [7]. Indeed, it is well known that approximately 70% of obstetric claims relate to events occurring during the delivery process [8]. Several physiological ocular changes are known to occur during pregnancy due to the activation of estrogenic and progesterone receptors [9,10]. A decrease in corneal sensitivity and intraocular pressure (IOP) and an increase in both corneal thickness and curvature or ptosis are common findings during pregnancy [9,10,11]. The early recognition and staging of the specific eye disorder represents a critical aspect in the management of pregnancy and in the decision regarding the appropriate mode of delivery. No universally accepted guidelines for obstetricians are available to guide clinicians in the decision on whether an elective CS should be advocated if the mother suffers from an ophthalmic pathology; therefore, we aimed to realize a state-of-the-art review of the available literature to provide the best evidence regarding ophthalmologic indications for CS.

## 2. Materials and Methods

A comprehensive literature search was conducted using MEDLINE, Embase, and the Cochrane Library from their inception through October 2024. The search strategy included the following keywords: “Caesarean section” OR “Cesarean section” OR “delivery” OR “pregnancy” AND “eyes” OR “eye disorders” OR “ocular disease” OR “diabetic retinopathy” OR “myopia” OR “retinal detachment” OR “glaucoma” OR “keratoconus”.

Studies were considered eligible if they described pregnancy management in women affected by an eye disorder, with insight into the mode of delivery.

The selection included original research articles, clinical guidelines, retrospective and prospective cohort studies, case–control studies, case reports, and expert consensus statements. No language restrictions were applied. Studies not focused on pregnancy management with insight into the mode of delivery were excluded. Three independent reviewers (P.Q., G.B., and G.C.) screened titles and abstracts for inclusion. As a narrative review, no formal guidelines were followed.

## 3. Results

A total of 8383 results were found. After the selection process, 38 studies were included [12,13,14,15,16,17,18,19,20,21,22,23,24,25,26,27,28,29,30,31,32,33,34,35,36,37,38,39,40,41,42,43,44,45,46,47,48,49,50]. Of these, 2 were case reports, 3 were case series, 1 was a guideline, 1 was a consensus statement, and 31 were peer-reviewed journal articles.

The reviewed articles were further categorized according to the ophthalmic pathology they were addressing. The following sections will provide a detailed analysis of the results that emerged from our research.

### 3.1. Myopia

Myopia is an axial eye elongation with a consequently more stretched retina. The risk of peripheral retinal tears is correlated with the severity of the disease. High-grade myopia (HGM), defined as a refractive error of at least −6.00 diopters or an axial length of ≥26 mm, is a major cause of visual impairment and blindness, especially in younger patients [11]. Myopia is the most common ophthalmological indication to deliver by CS [12]. For a long time, gynecologists, obstetricians, and ophthalmologists shared the opinion that myopia (especially HGM) was a contraindication for spontaneous vaginal delivery due to concerns about retinal detachment [13]. The decision to perform an instrumental delivery (vacuum extraction and forceps delivery) or a CS, in an attempt to shorten the second stage of labor and protect women’s eyes from injuries, was based mainly on the absolute value of refractive error in a given pregnant woman, irrespective of any additional retinal abnormalities [13]. This concern was formally addressed as early as 1972 by Atassi et al., who recommended a CS or an operative vaginal delivery to women with myopia of greater than −4.0 diopters, aiming to prevent a rise in the intraocular pressure [13].

However, the available literature contains no reports describing retinal detachment in the presence of a HGM after spontaneous vaginal delivery. In detail, a study by Neri et al. comprised 50 myopic women (−4.5 to 15 diopters) examined by ophthalmologists, who were experts in retinal disorders, before and after delivery. Despite the identification of retinal degenerative changes, including lattice-like degeneration (17/50 (34%)) and retinal breaks (11/50 (22%)), along with myopia, no retinal changes were reported after delivery [14]. A similar study conducted by Prost M. reported no progression of retinal changes after birth [15]. A smaller study by Landau D. et al. examined 10 women (19 deliveries) with variable retinal changes: 8/10 (80%) had lattice-like degeneration changes, 8/10 (80%) experienced retinal tears requiring laser treatment, and 6/10 (60%) underwent a surgical repair of a retinal detachment before pregnancy. Again, the postpartum follow-up examination of the retina did not show any significant changes when compared with the antenatal ocular findings [16].

Going back to the pathophysiology, it should be considered that a rise in intraocular pressure (IOP) during the second stage of labor is associated with a vitreous body being pressed against the retina, which reduces the risk of retinal tears and retinal detachment rather than increasing such risk [13,16]. Moreover, recent studies suggest that exhalation against a closed airway, which occurs during the Valsalva maneuver, leads to a choroid increase. This expansion could function as a protective mechanism, rather than a trigger for retinal detachment. Thus, the long-standing assumption that increased IOP during labor directly contributes to retinal detachment has been questioned [17].

A more specific chapter regarding the mode of delivery in the case of an HGM is the one about cases characterized by the presence of choroidal neovascularization (CNV). Myopic patients with concomitant CNV in the macular region carry an elevated risk of retinal hemorrhage during the second stage of labor and, subsequently, a possibility of a non-reversible sudden deterioration of visual acuity [18]. However, there is no evidence that such conditions may worsen after vaginal delivery [18,19], except from only one case report [19], in which macular hemorrhage developed postpartum. Notably, this case was not attributed to macular CNV but rather to macular staphyloma with lacquer cracks. In 2018, the Polish Society of Gynecologists and Obstetricians published the only available guideline about the mode of delivery in the presence of ophthalmic pathology, recommending a CS in women with HGM only in the presence of macular CNV [20]. However, this recommendation comes from a 2017 consensus, which does not include any cited literature references [21].

In conclusion, for those women that underwent refractive surgery due to HGM, no evidence is available regarding the best way to deliver, and ophthalmologists themselves have demonstrated the opposite position regarding this circumstance, but according to a recent survey in the case of a history of refractive surgery, ophthalmologists recommend a vaginal delivery over cesarean section twice as much as their gynecologist peers [22,23]. All pregnant women affected by HGM should undergo a complete ophthalmological examination at the first and then at the third trimester of pregnancy, including the study of the macula and peripheral retina for any anomalies (e.g., choroidal neovascularization). In the presence of degenerative changes, or retina tears predisposing to retinal detachment, laser coagulation should considered no later than one month before birth, aiming to prevent potential complications [20,21,22,23,24].

### 3.2. Retinal Detachment

Retinal detachment (RD) is a serious ocular condition. It is defined as “rhegmatogenous” if induced by underlying conditions and “not rhegmatogenous” when no underlying conditions have been detected. The latter is often related to severe obstetric complications, such as pre-eclampsia/eclampsia. In clinical ophthalmology or obstetric practice, retinal detachment (RD) commonly refers to rhegmatogenous retinal detachment (RRD) [25]. The term “rhegmatogenous” derives from the Greek “reghma”, or fracture, and is characterized by the presence of a full-thickness retinal break. This break is held open by vitreoretinal traction that allows the accumulation of liquefied vitreous into the potential space between the retinal pigment epithelium (RPE) and the neurosensory retina. This is by far the most common presentation of RD. This condition has an age-related prevalence. It occurs in approximately 11% of people in their sixties and increases to 46% of people in their 80s [26]. However, in individuals with myopia, RRD often appears earlier. This is because up to 60% of retinal detachments are associated with degenerative changes in the equatorial region of the retina, particularly in myopic eyes. Studies suggest that, worldwide, retinal detachments occur in 6–9.5% of the population, but in people with myopia of greater than −3.0 diopters, this number increases to 14%, compared with just 4.3% in individuals with emmetropia (normal vision) [26,27]. Two UK surveys suggest obstetricians may recommend an assisted vaginal delivery with forceps, vacuum extraction, or a cesarean section to women who had surgery for RRD. This decision is due to fear of recurrent retinal re-detachment [28].

Despite this elevated risk for retinal detachment, the literature does not support the idea that vaginal delivery is associated with a higher incidence of retinal detachment or the development of new peripheral retinal degenerations, which could act as precursors to detachment. This is true even in cases where women have a history of vitreoretinal surgery [28,29]. Particularly, the study by Landau D. et al. supports such a conclusion, wherein almost the entire study population (women who experienced retinal detachment during the third trimester of pregnancy and underwent surgical treatment) did not experience any ocular complication after vaginal delivery [16]. Additionally, there are several studies on small populations of women who experienced safe vaginal delivery after surgically treated RRD (pars plana vitrectomy or scleral buckling) [30].

Despite long-standing concerns, current evidence suggests that in patients with previous retinal detachment, vaginal delivery is safe. However, in cases where significant risk factors for retinal detachment are present, such as HGM, it is crucial to recommend women undergo a detailed ophthalmic examination before or during pregnancy. This suggestion is aimed at identifying conditions that might require treatment, such as laser photocoagulation, to prevent complications during delivery. By taking these precautionary steps, women with myopia or other retinal conditions can safely undergo vaginal delivery, minimizing the need for unnecessary surgical interventions [31,32].

### 3.3. Diabetic Retinopathy

Diabetic retinopathy (DR) is a common microvascular complication of diabetes. It is a leading cause of vision impairment in people aged 20–64 years, which overlaps with the childbearing years for women. Indeed, the prevalence of diabetic retinopathy in patients with type 2 diabetes is 14% during pregnancy [33,34]. Pregnancy is an independent risk factor for DR progression; indeed, the increase in progesterone levels results in increased synthesis of vascular endothelial growth factor, which plays a key role in the development and progression of DR due to its effect on vascular wall permeability and stimulating neoangiogenesis [12,22]. During Valsalva maneuvers, when the systemic blood pressure increases, a sudden escalation in the intraocular venous pressure also occurs, with a potential subsequent rupture of superficial retinal capillaries, especially in long-standing DR, where the retinal vessels are more fragile [35,36,37]. Also, diabetic compensation status correlates with the risk of DR progression, and conditions such as diabetes duration, onset timing, elevated first-trimester HbA1C levels, persistent poor glycemic control, chronic hypertension, and nephropathy with proteinuria are all contributing factors to the risk of progression from non-proliferative to proliferative DR [33,34]. The management of DR during pregnancy poses many challenges, including the potential of DR’s rapid progression, difficulties for women attending a substantial number of medical appointments, and fetal contraindications for medical treatments usually adopted to treat DR complications, such as vitreous hemorrhage. This last complication may occur in the proliferative phase of DR [34,35,36,37]. Recent evidence also suggests that the presence of DR is associated with an increased risk of obstetric complications (pre-eclampsia and preterm birth) and CS [38]. International guidelines suggest that pre-gestational diabetic women without symptoms of DR should undergo an ophthalmological examination every trimester, whereas, in the case of already diagnosed pre-gestational DR, a monthly check is advised during pregnancy. Laser coagulation treatment may be considered for pregnant women with proliferative DR [12,22,23,37,38,39]. Therefore, a vaginal delivery, if not otherwise contraindicated, should be considered in women with stable retinal status throughout their pregnancies without evidence of proliferative DR [39]. On the contrary, in the case of proliferative DR, previous vitreous hemorrhage, or the presence of tractional retinal detachment that develops or progresses throughout pregnancy, due to the intravitreous bleeding risk from pathological vessels, a CS should be offered [12,22,37,39].

Continuous ophthalmological monitoring throughout pregnancy is crucial in managing the condition effectively and ensuring the best outcomes for both mother and baby. Regression of diabetic retinopathy and spontaneous vision recovery often occur in the postpartum period [12,22,23,36].

### 3.4. Glaucoma

Glaucoma, a condition in which there is a build-up of fluid in the eye, which presses on the retina and the optic nerve, is rarely of concern during pregnancy, since it usually does not affect women in their reproductive age [40]. However, in the case of pre-gestational glaucoma or ocular hypertension (OHT), pregnancy-related changes in the IOP may significantly impact the eye disorder and, therefore, require careful management [9,10].

Pregnancy often induces a natural reduction in IOP, which is the critical risk factor for the progression of glaucomatous optic nerve damage. This reduction in IOP is more pronounced in women with OHT. Studies have shown that during pregnancy, IOP decreases by approximately 24.4% in women with OHT, compared with a 19.6% decrease in healthy pregnant women [40,41]. This hypotensive effect may be due to an increased outflow of aqueous humor, even though its production remains unchanged. Notably, this lower IOP can persist for up to six months postpartum, offering some temporary relief for glaucoma patients during and after pregnancy [41]. Pregnancy-related hypertension also influences the IOP. In general, pregnant women with hypertension experience only a slight IOP increase (about 0.5 mmHg) in the third trimester, while those with pre-eclampsia may see a more significant rise, with the IOP increasing by as much as 3.5 mmHg during the peripartum period [40].

The situation changes dramatically during labor, especially during the second stage when pushing occurs. During uterine contractions in the first stage of labor, the average IOP increases by around 4 mmHg [12,17]. However, during the pushing phase (associated with the Valsalva maneuver), the IOP can rise by approximately 12 mmHg. These fluctuations in pressure, though temporary, can be significant for patients with advanced glaucoma or those already at high risk of optic nerve damage [13,14,15,16,17]. The IOP fluctuations during labor have led to concerns about the potential for retinal ganglion cell loss, which can contribute to the progression of visual field loss in patients with glaucoma [42,43]. These concerns in the group of women with advanced glaucomatous optic atrophy resulted in the recommendation of an assisted vaginal delivery (forceps or vacuum extraction) [22]. Such interventions may reduce the number of high-pressure spikes during the pushing phase. Nevertheless, glaucoma has been reported to represent only 5% of cesarean sections performed for ophthalmic pathology, suggesting that severe complications due to labor-related IOP increases are rare [40,44]. Managing IOP in pregnant women with glaucoma requires a careful balance between effective treatment and safety for both mother and fetus. As IOP is naturally reduced during pregnancy, aggressive treatment is rarely needed and should be performed with drugs devoid of negative effects on the fetus. Medications like prostaglandin analogs are typically avoided due to potential risks, while beta-blockers and alpha agonists are used with caution. If pharmacological treatment is ineffective, then laser trabeculoplasty should be considered as a safer, non-invasive option during pregnancy. Only for the more severe cases where medications and laser treatments fail to control IOP, surgical interventions like trabeculectomy may be necessary, though these are typically reserved for the postpartum period unless absolutely required. Regular monitoring and individualized treatment plans are crucial, considering the fluctuating nature of IOP during pregnancy and the postpartum period [12,22,23,45,46,47,48].

While vaginal delivery remains safe for most pregnant women with glaucoma or OHT, certain considerations should be made for those with advanced disease. The physiological pushing technique, which is less forceful than the Valsalva maneuver, is recommended for minimizing IOP spikes during delivery.

The decision on the mode of delivery should be made on a case-by-case basis, depending on the severity of the glaucoma. A pivotal aspect of the management of glaucoma during pregnancy is ensuring regular ophthalmologic follow-ups to monitor IOP and optic nerve health. In some cases, obstetricians may recommend performing a Valsalva maneuver early in pregnancy as a provocation test to assess the individual IOP response and predict how the patient might tolerate the pressures of labor [40].

After delivery, IOP often remains lower than pre-pregnancy levels for up to six months, offering a protective effect for women with glaucoma. This extended period of lower IOP may reduce the immediate risk of progression of glaucomatous damage. However, women should continue regular monitoring by their ophthalmologist during this time to ensure no further complications arise.

In summary, pregnancy generally exerts a beneficial effect on IOP, reducing pressure and temporarily alleviating some of the risks associated with glaucoma. However, labor presents a unique challenge due to the significant fluctuations in IOP that occur during contractions and pushing. For most women with glaucoma or OHT, vaginal delivery is safe, though patients with advanced disease may require closer monitoring and possibly an assisted vaginal delivery or a CS depending on the specific condition to minimize the risk [12,22,23,45,46,47,48].

### 3.5. Keratoconus

Keratoconus is a progressive, bilateral, and usually asymmetric disorder causing progressive thinning and conical protrusion of the cornea. According to existing evidence, a progression of keratoconus takes place during pregnancy [49]. Particularly, Bilgihan K. et al. showed that pregnancy-related hormonal changes may affect corneal biomechanics. Elevated estrogen levels and increased matrix metalloproteinase (MMP) activity, along with reduced tissue inhibitors of MMPs (TIMPs), may weaken the cornea, leading to ectasia progression during pregnancy. Relaxin, a hormone known for collagen breakdown during pregnancy, may also contribute to this process. Corneal cross-linking, a treatment to halt keratoconus progression, might be more frequently recommended for women with keratoconus who are planning a pregnancy [50]. Moreover, according to the Polish Society of Gynecologists and Obstetricians, delivery via CS is indicated in patients with advanced or acute keratoconus [12,22]. Meanwhile, previous studies, such as the Collaborative Longitudinal Evaluation of Keratoconus, found no connection between pregnancy hormone status and keratoconus [50]. However, further studies are required to elucidate the clinical correlation between keratoconus and pregnancy.

## 4. Conclusions

The choice of the appropriate mode of delivery for pregnant women affected by an ophthalmic pathology such as myopia, retinal detachment, diabetic retinopathy, glaucoma, and keratoconus should be individualized based on the severity of the condition and the associated risks. For HGM, current guidelines recommend CS only in the presence of macular CNV, therefore, all pregnant women with HGM should undergo a comprehensive ophthalmologic evaluation during pregnancy, aiming to exclude the presence of CNV. The history of retinal detachment generally does not require a CS, as most studies indicate that vaginal delivery is safe even after retinal surgery. In cases of diabetic retinopathy, particularly proliferative retinopathy, CS may be recommended to prevent complications such as vitreous hemorrhage during labor. But women with stable non-proliferative diabetic retinopathy can undergo a safe vaginal delivery. Regarding glaucoma, pregnancy often leads to a natural lowering in IOP, making vaginal delivery the first option in most cases. However, in the presence of advanced glaucoma, where labor-induced IOP spikes could be harmful, CS or assisted vaginal delivery may be taken into account. Finally, in the presence of keratoconus, whose progression during pregnancy requires close monitoring, a CS may be advised in severe cases, aiming to prevent further corneal damage. In conclusion, based on the limited available evidence, an elective CS may be considered on a case-by-case basis in the presence of highly specific ophthalmic conditions, such as high-grade myopia with evidence of subretinal neovascularization, proliferative diabetic retinopathy, advanced glaucoma, or advanced keratoconus. It should be noted that these conditions are rare among women of childbearing age. Considering the potential short- and long-term implications of a CS, and in line with the current World Health Organization recommendations, this surgical procedure should be reserved only for those cases with a recognized indication. Due to a paucity of data in the existing literature, further prospective randomized controlled trials are needed to strengthen the quality of evidence.

**Figure 1:**
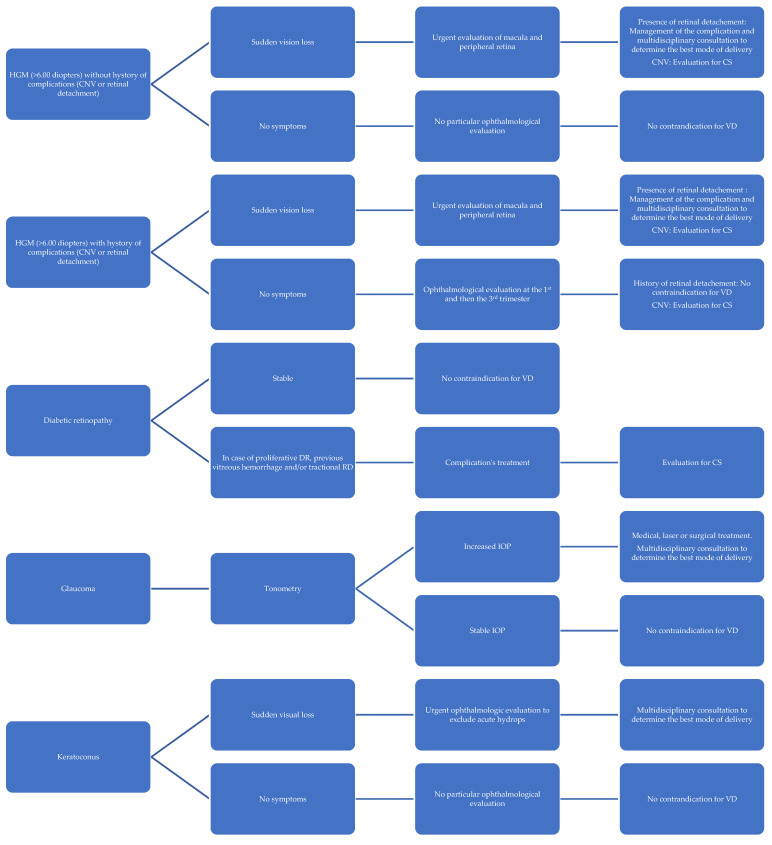
Ophthalmological indication for cesarean section: suggested management protocol.

A management protocol based on the findings of our narrative review is presented in Figure 1.

## Data Availability

The raw data supporting the conclusions of this article will be made available by the authors on request.

## Abbreviations

HGM: high-grade myopia; CNV: choroidal neovascularization; VD: vaginal delivery; DR: diabetic retinopathy; RD: retinal detachment; CS: cesarean section; IOP: intraocular pressure.
